# Microbial Communities in Bioswale Soils and Their Relationships to Soil Properties, Plant Species, and Plant Physiology

**DOI:** 10.3389/fmicb.2019.02368

**Published:** 2019-11-20

**Authors:** Olivia L. Brodsky, Katherine L. Shek, Devin Dinwiddie, Sarah G. Bruner, Aman S. Gill, Jessica M. Hoch, Matthew I. Palmer, Krista L. McGuire

**Affiliations:** ^1^Department of Environmental Science, Barnard College, Columbia University, New York, NY, United States; ^2^Department of Biology, Institute of Ecology and Evolution, University of Oregon, Eugene, OR, United States; ^3^Department of Ecology, Evolution and Environmental Biology, Columbia University, New York, NY, United States; ^4^Department of Environmental Science, Policy, and Management, University of California, Berkeley, Berkeley, CA, United States

**Keywords:** combined sewage overflow, urban green infrastructure, bioswale, plant functional traits, soil microbes, plant-soil relations

## Abstract

Bioswales and other forms of green infrastructure can be effective means to reduce environmental stresses in urban ecosystems; however, few studies have evaluated the ecology of these systems, or the role that plant selection and microbial assembly play in their function. For the current study, we examined the relationship between plant transpiration rates for five commonly planted herbaceous species in three bioswales in New York City, as well as bioswale soil microbial composition and soil chemistry. Soils were sampled near individual plants, with distinction made between upper (bioswale inlet) and lower slopes (bioswale outlet). We found high variation in transpiration rates across species, and that *Nepeta × faassenii* was the highest conductor (13.65 mmol H_2_O m^–2^s^–1^), while *Panicum virgatum* was the lowest conductor (2.67 mmol H_2_O m^–2^s^–1^) (*p* < 0.001). There was significant variation in percent N of leaves and soil, which did not relate to the higher water conductance in bioswales. Significantly higher C, N, and water content on the high end of bioswale slopes suggest storm water run-off is mostly absorbed on the inlet side. Bacterial and fungal communities were significantly clustered by bioswale and by plant species within each bioswale implying there are micro-environmental controls on the soil microbial composition, and that plant composition matters for microbial assemblages within bioswales. Plants with higher transpiration rates were associated with greater fungal and bacterial diversity at the level of the bioswale and at scale of the individual plant, suggesting a possible link between plant physiological traits and soil microbial communities. These data suggest that the specific plant palette selected for planting bioswales can have deterministic effects on the surrounding microbial communities which may further influence functions such as transpiration and nutrient cycling. These results may have implications for bioswale management to improve urban water quality and reduce stress on sewage systems after storm events by revising plant species palette selection based on the functional consequences of plant-microbial associations in engineered green infrastructure.

## Introduction

Cities with older infrastructure that combine both sanitary, industrial, and storm sewers are at risk of overwhelming the capacity of these sewer systems during wet weather events, which trigger combined sewer overflows (CSOs) into nearby waterways (Burian et al., 2000). These CSOs can be triggered by a single precipitation event, and are related to significant economic, human health, and environmental problems (Lee and Bang, 2000; Marsalek and Rochfort, 2004; Gasperi et al., 2010). The frequency of CSO events is expected to increase substantially in coming years, as models predict the proportion of the world urban population to reach 66% by 2050, which is nearly double the urban population from 1950, putting great strain on the quality of water resources (Vorosmarty et al., 2000; Grimm et al., 2008). One common strategy for mitigating the frequency and velocity of CSO events is the construction of green infrastructure, which are pervious, vegetated areas in cities that capture precipitation and reduce the loading in combined sewer systems from runoff derived from impervious surfaces (Carson et al., 2013). Bioswales, which are permeable surfaces containing trees and other vegetation that are engineered to divert stormwater runoff from the street and into the soils, have become particularly prominent in urban development initiatives to reduce CSO events (Anderson et al., 2016; Woznicki et al., 2018). However, few studies have evaluated the ecology of these systems, so whether or not particular plant palettes are more or less effective for the multi-functionality of their benefits remains uncertain.

While plants are the prominent biological design feature of bioswales, microbes that reside within and around the plants are a relatively unexplored aspect of bioswales that have the potential to significantly increase green infrastructure functionality (McGuire et al., 2015; Fulthorpe et al., 2018). Plants and soil play critical roles in storm-water absorption and overall bioswale effectiveness (Xiao and McPherson, 2011), and plants rely on soil microbes to gain access to nutrients and water that otherwise would not be available (Auge, 2001; van der Heijden et al., 2008). As an extended component of a plant’s phenotype, microbes have the potential to facilitate plant resilience to frequent drying and wetting events that occur in bioswale soils, which would enhance plant survival and longevity in these systems. In addition, pollutants and excess nitrogen can be considerably high in bioswale soils due to the influx of stormwater (Shetty et al., 2018), and there is evidence that high abundances of microbial genes involved in contaminant degradation are expressed in bioswale soils (Gill et al., 2017). Previous studies have found that bioswale plants vary in their efficiency at pollutant removal (Read et al., 2008), and these effects are likely microbially mediated (Friesen et al., 2011). Plant-associated microbes may also influence plant physiological responses such as transpiration rates (Li et al., 2018), which have the potential to directly affect the quantity of water movement in bioswales.

In this study, we evaluated plant-associated microbial communities in bioswales in New York City, and measured relative rates of transpiration in the plants they were associated with. We chose five herbaceous target plant species that are common in NYC bioswale plant palettes and hypothesized that (1) each plant species would host unique soil microbial assemblages; (2) water conductance of the five-plant species both within and across bioswales would reveal species-specific patterns; and (3) variation in plant physiology would correlate with variation in soil microbial composition. These results present potential improvements for the design of bioswales for optimal hydrology and other desired functions such as pollutant removal.

## Materials and Methods

### Study Site

Three bioswales in Rego Park Queens, New York, were chosen that were all established in 2013, 3 years prior to sampling. The bioswales were chosen based on their similar planting design to keep the distribution of species controlled. Five native plant species, *Aronia melanocarpa* (Rosaceae), *Eupatorium dubium* (Asteraceae), *Nepeta × faassenii* (Lamiacae), *Panicum virgatum* (Poaceae), and *Spiraea nipponica* (Rosaceae), were selected as our focal study species, as these are commonly included in bioswale plant palettes in this region, and all form relationships with arbuscular mycorrhizal (AM) fungi. Soils from the bioswales examined in this study were derived from the same homogenous mixture installed by the same contractor, and have not been modified since planting.

### Plant Physiology Methods

Stomatal conductance (mmol m^–2^ s^–1^) was measured for each of the five-plant species using a steady state leaf porometer (Decagon Model SC-I). Four measurements were taken from each plant species within each of the three bioswales between 10:00 and 14:00, on September 17, 2016. A portable weather station recorded photosynthetically active radiation, wind speed, temperature and the transpiration rates.

To solve for the Vapor Pressure Deficit (VPD), we use the following equation:

$${\text{VP}}_{\text{leaf}}-{\text{VP}}_{\text{air}}=\mathrm{VPD}\,\mathrm{kPa}$$

To relate transpiration (E) to the VPD, we use the following equation:

$$\text{E}=\left[\frac{\text{VPD}}{\text{BP}}\right]\times g_{\text{sw}}$$

where E is the transpiration rate in mmol H_2_O m^–2^s^–1^, VPD is the vapor pressure deficit in kPa, BP is the barometric pressure in kPa (101.3 at sea level), and *g*_sw_ is the stomatal conductance to water vapor (Lambers et al., 2008).

### Soil Collection and Soil Physicochemical Analyses

Soil samples were collected on September 17, 2016 in Rego Park, Queens, New York. Using a soil corer (2.5 cm × 10 cm), soil samples were collected at the base of each plant for evaluation of species-specific soil microbial assemblages. Each bioswale was constructed with a slope to facilitate entry storm water to inlet, or high end of the slope, and exit of water through the low end, or outlet. Two individuals of each plant species were identified that were closest to the high and low end of each bioswale, and these were selected as our focal individuals for sampling. Soil adjacent to four individuals of each target species were sampled within each bioswale. Individual soil cores were passed through sterile 2.0 mm sieves and placed into cryovials that were flash frozen in liquid N on-site for subsequent molecular analysis. The remaining soil from each core was placed in a sterile Whirl-Pak bag (Forestry Suppliers, Jackson, MS, United States) for soil physicochemical analyses (specifically, percent water content, pH, and total carbon and nitrogen). The Whirl-Pak bags and cryovials were frozen at −80°C on the same day of collection. Soil pH was analyzed with a gas electrode using a 1:2 ratio with water. Total C and N content was quantified by combustion using an Elementar Vario-Macro CNS analyzer at the Lamont Doherty Earth Observatory (Palisades, NY, United States). To calculate volumetric water content, 5 g of wet soil was weighed and recorded, dried at 105°C for 24 h, and reweighed for each sample.

### Microbial Analyses

Total DNA from soil was extracted from approximately 0.25 g per sample using MoBio PowerSoil DNA extraction kits (MO BIO Laboratories Inc., Carlsbad, CA, United States). Genomic DNA was amplified using the ITS1-F and ITS2 primers for fungi (McGuire et al., 2013) and the 515-F and 806-R primer pair for the 16S rRNA gene in bacteria (Caporaso et al., 2011). PCR was conducted in 25 μL reactions containing 10 μL H_2_O, 12.5 μL GoTAQ 2X MM mix (Promega, Madison, WI, United States), 0.5 μL of both the forward and reverse Illumina-barcoded primers, and 1 μL of genomic DNA. PCR cycles were performed at 94°C for 3 min, 35 cycles of 94°C for 45 s, 50°C for 60 s, 72°C for 90 s, then 10 min at 72°C. All the PCR reactions were run in duplicate, and visualized using gel electrophoresis. Successful reactions were quantified using a spectrofluorometer, pooled, and sent for Illumina MiSeq paired-end sequencing. Reads were processed using the UPARSE pipeline software pipeline (Edgar, 2013) and sequences were clustered at 97% similarity to identify operational taxonomic units (OTUs). DNA reads were de-multiplexed and filtered to eliminate low-quality reads with a maximum *e*-value of 1, leading to sequences with a maximum average of 1 base error per sequence, using UPARSE (Edgar, 2013). Taxonomic assignments were determined using the Ribosomal Database Project classifier. Sequence abundances were rarefied to 3,450 sequences per sample for fungi and 12,900 sequences per sample for bacterial data. The OTUs that were not assigned to a taxonomic group were filtered out.

### Statistical Analyses

Statistical comparisons of plant species’ transpiration data as well as variation in microbial community composition with various physicochemical parameters were conducted using the R language and environment for statistical computing (R Core Development Team, 2011). To visualize patterns of fungal and bacterial clustering across bioswale and plant species, we used non-metric multidimensional scaling (NMDS) with the ordinate function in phyloseq (McMurdie and Holmes, 2013) with Bray–Curtis distance metrics. Permuted analysis of variance (PERMANOVA) was run to test clustering significance using the Adonis function in the Vegan package (Oksanen et al., 2017). Relationships between microbial diversity and collected metadata were investigated by calculating Pearson’s correlations using the rcorr function in the Hmisc package (Harrell, 2016). For this analysis, OTU tables were transformed to the square root relative abundance using the decostand function in Vegan (Oksanen et al., 2017) and OTUs that were significantly correlated (*p* ≤ 0.05) with log transpiration rate were extracted for each plant species and analyzed for correlations with other metadata variables. *P* values were derived using Anova and Tukey’s Honestly Significant Difference (HSD) tests (Abdi and Williams, 2010). All permutation tests were performed with 999 permutations and figures were prepared using ggplot2 (Wickham, 2016). Functional guilds were extracted for the fungal OTUs using FUNGuild v1.1 (Nguyen et al., 2016). Guilds aligning to saprotrophs, plant pathogens, and AM fungi were separately analyzed for richness at the genus level.

## Results

### Water Conductance

**FIGURE 1:**
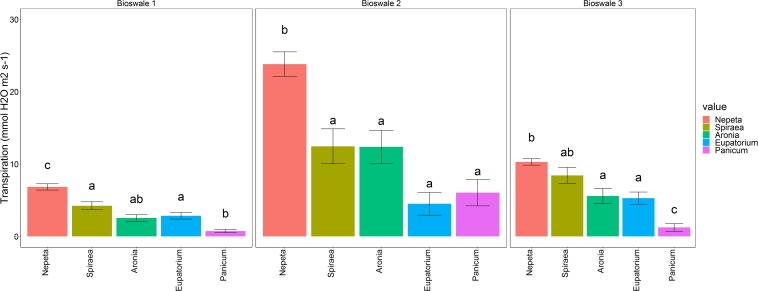
Average transpiration rates of the five target plant species separated by bioswale site (mean ± 1 SE). Bars with different letters are significantly different at a level of *p* < 0.05.

Transpiration readings show *Nepeta* as the highest water conductor with values averaging to 13.65 mmol H_2_O m^–2^s^–1^. *Panicum* conducted the least water with 2.67 mmol H_2_O m^–2^s^–1^, and intermediate similar results for *Aronia*, *Eupatorium*, and *Spiraea*, which averaged ∼6.41 mmol H_2_O m^–2^s^–1^ (Figure 1; *p* < 0.001). These patterns among species were relatively consistent, despite some variation across bioswales.

### Soil Physiochemical Analyses

Percent N varied significantly across bioswales (*p* < 0.05), but not significantly by species, and there was no significant difference in soil C variation. However, the slope (high or low) of the bioswale showed significant variation in percent soil N, C, and water content (*p* < 0.05). The inlet end of the slope had nearly 1.6 times more C, double the N, and nearly 1.5 times more water content. Percent water content did not vary significantly across bioswales or species. The C:N ratios and soil pH were not significantly different in slope or across species.

### Microbial Analyses

For the bacterial community amplicon-based sequencing, OTU reads were rarified to an even depth of 6200 and OTUs that were unclassified at the Domain level were removed, leaving a total of 16619 identified bacterial OTUs. The fungal OTU reads were rarified to an even depth of 1400 and OTUs that were unclassified at the Domain level were removed, leaving a total of 3954 identified fungal OTUs.

**FIGURE 2:**
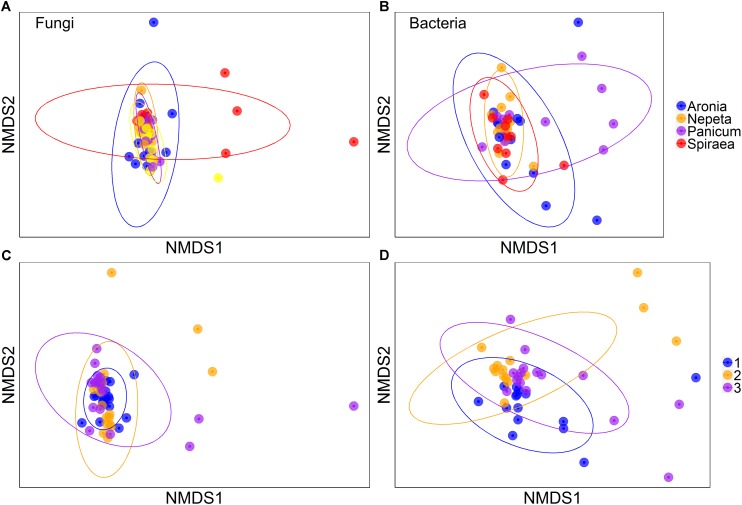
Non-metric multidimensional scaling of fungi across plant species **(A)** and bioswale **(C)** and also of bacteria across plant species **(B)** and bioswale **(D)**. Figure represents dissimilarity as the Bray–Curtis metric and the ellipses signify 95% confidence intervals.

Multiplicative PERMANOVA tests found significant effects of plant species, bioswale location, and slope position on bacterial community composition (Figure 2; *p* < 0.05). There were also significant interaction effects between plant species and bioswale location, plant species and slope position, as well as bioswale location and slope position (*p* < 0.05). Similarly, plant species and bioswale location had a significant effect on fungal community composition (*p* < 0.05) and an interaction effect between plant species, bioswale location and slope position was also found (*p* < 0.05).

**FIGURE 3:**
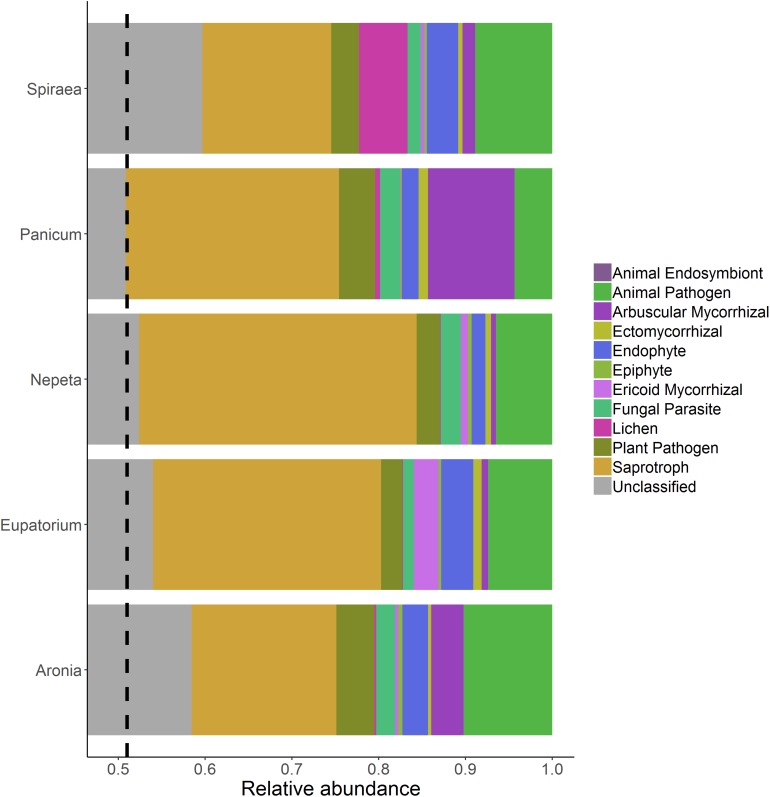
Relative abundance of fungal functional groups from soils collected near the individual plant species listed on the left side of each bar. The dashed line references the start of the lowest unclassified grouping because the *x*-axis does not start at zero.

**FIGURE 4:**
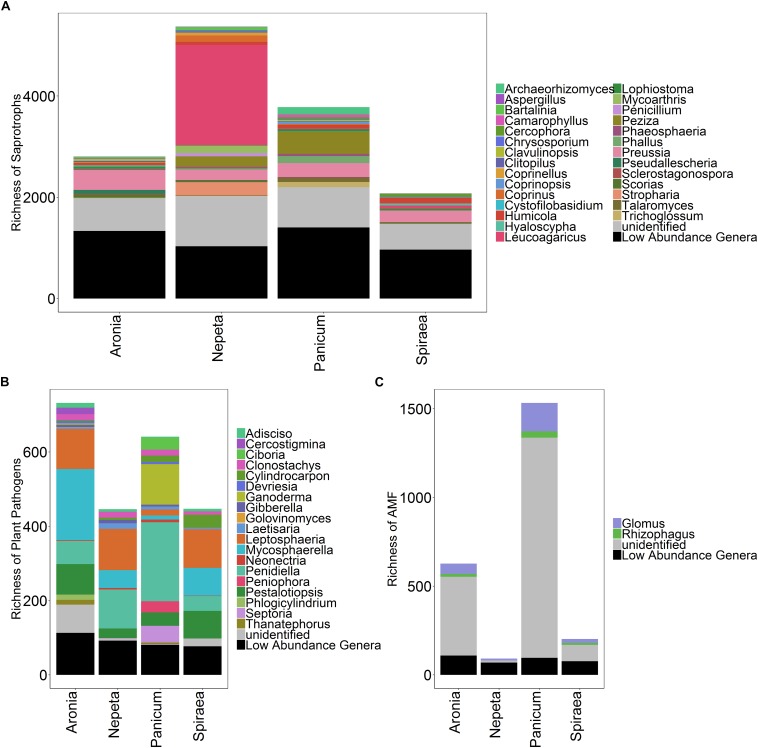
Richness of saprotrophs **(A)**, plant pathogens **(B)**, and arbuscular mycorrhizal fungi **(C)** isolated from soils collected near the five target plant species.

Functional guilds of fungi were assigned with a total of 1994 of the 3945 fungal OTUs assigned to functional guilds (Figure 3). There were no significant differences in the relative abundances of individual functional groups across plant species; however, soils collected beside the *Panicum* species had significantly higher relative abundances of AM fungi than the other four plant species (Figure 4).

**FIGURE 5:**
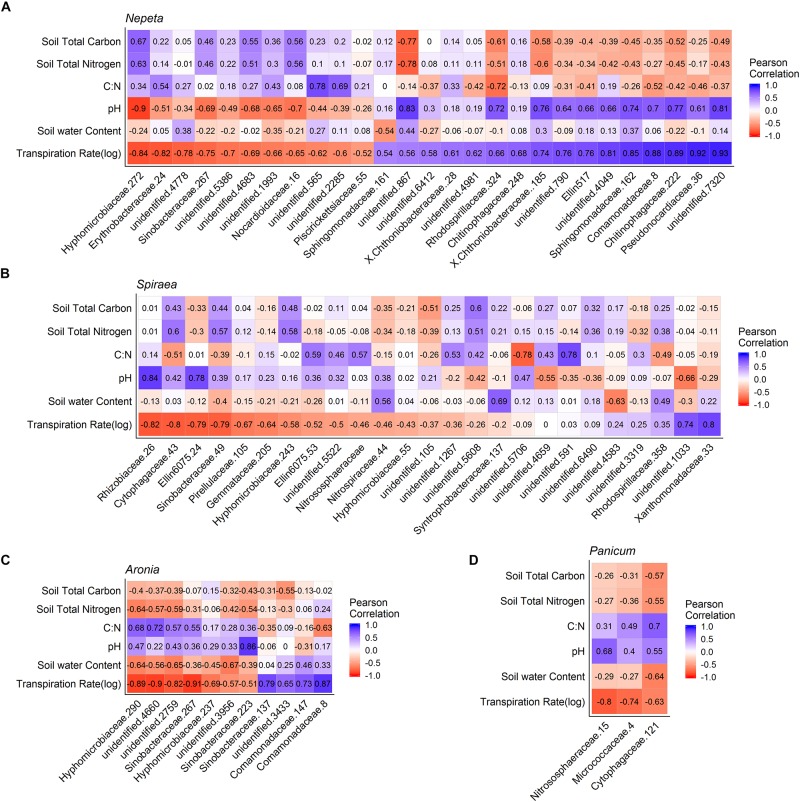
Pearson correlation matrix of bacteria OTUs that were significantly correlated with the plant transpiration for *Nepeta* **(A)**, *Spiraea* **(B)**, *Aronia* **(C)**, and *Panicum* **(D)**. *Eupatorium* was not included due to low sample sizes.

**FIGURE 6:**
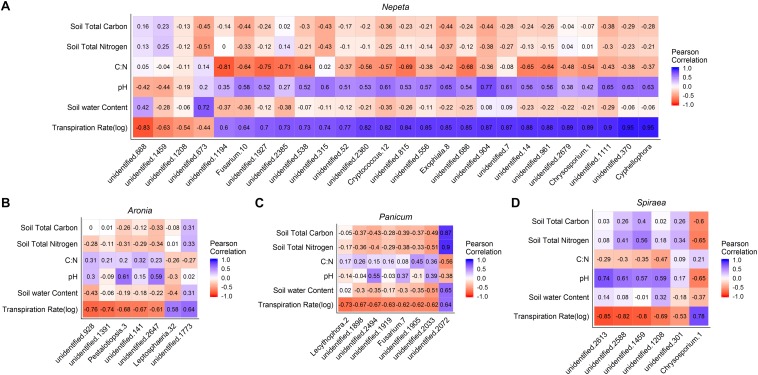
Pearson correlation matrix of fungal OTUs that were signficantly correlated with the plant transpiration for *Nepeta* **(A)**, *Aronia* **(B)**, *Panicum* **(C)**, and *Spiraea* **(D)**. *Eupatorium* was not included due to low sample sizes.

To examine correlations between microbial communities, soil physicochemical properties and plant transpiration rates, Pearson’s correlation metrics were calculated for microbial communities by each of the five plant species sampled. To eliminate any spurious correlations we chose to only examine OTUs that had a correlation (±) with at least 10 sampled plants of the same species for bacteria and at least four sampled plants of the same species for fungi. Following that correction, a total of 66 bacterial OTUs and 47 fungal OTUs were significantly correlated (*p* < 0.05) with plant transpiration rate across plant species (Figures 5, 6). Since too few taxa were detected in *Eupatorium*, that species was excluded from the final analysis. Certain fungal taxa in the Comamondaceae, and bacteria taxa in the genus Chrysosporium and were found associated with higher transpiration rates within and across plant species (Figures 5, 6).

## Discussion

Bioswales play an integral role in mitigating the incidences of storm water overflow events, and this study is among the first of its kind to detect relationships between the physiological traits of plants, the abiotic properties of soils, and the community composition of soil microbes that occupy these engineered ecosystems. In non-engineered ecosystems, microbial communities tend to be influenced first by climate, then by soils, and finally by biotic factors such as plant species (Fierer, 2017). We found strong separation of microbes across bioswales that was related to soil physical and chemical properties, and possible microclimatic effects that were not measured in the current study. At the local scale, microbial communities segregated according to which plant species they were collected near, likely in response to the specific aboveground and belowground traits of those plant species (Barberán et al., 2015; Leff et al., 2018; Zhalnina et al., 2018). The functional consequences of these specific plant-microbial assembly patterns were not assessed in our study, but may influence the capacity of bioswale soils to cycle nutrients and metabolize stormwater-derived pollutants such as hydrocarbons. In another study of bioswales in New York City, RNA sequencing revealed high expression levels of microbial genes involved in the decontamination and degradation of a suite of organic pollutants associated with stormwater (Gill et al., 2017). Together, the results from the current study and those of the Gill et al. (2017) study suggest that the location of the bioswale and the particular plant palette selected for planting, can ultimate influence the assembly and function of the soil microbial communities in these engineered ecosystems.

The significant interspecific variation among our five target plant species in two traits related to water movement and transpiration rates, indicates that species selection in bioswale designs will play an important role in determining the quantity of water that is captured and diverted from combined sewer systems. Specifically, we found that *Nepeta* (Lamiaceae), a widely used herbaceous plant in NYC green infrastructure, maintained the highest rates of water conductance across our bioswale study sites and may be beneficial to include and maintain in bioswale plant palettes. By contrast, *Panicum* (Poaceae) consistently had the lowest transpiration rates, probably due to its C4 physiology traits adapted for water conservation. While C4 plants may be beneficial for climates that experience frequent drought, they may not be the best choice for plant palettes in cities such as New York, where precipitation occurs frequently. Our study focused on the herbaceous understory plants of the bioswales, but other life forms such as trees and shrubs are often planted in these types of green infrastructure installations, and would be beneficial to examine in future studies to get integrated estimates of plant water movement from soils. One study of bioswale trees near Chicago found similar results to ours in that tree species varied widely in their transpiration and growth rates, and had dramatic differences on estimated bioswale water budgets (Scharenbroch et al., 2016). Bioswale plant choice can also influence the quantity of pollutants filtered from diverted stormwater (Read et al., 2008), although plant-associated microbes likely mediate those effects. Accounting for context-dependent plant trait variation across different urban climates and soils will be important to identify which plant palette configurations will ultimately contribute to more dimensions of the multifunctional ecosystem services of bioswales.

We found significant relationships between plant transpiration rates and the abundance of several groups of fungi and bacteria in the soils, which could result from two alternative explanations: (1) transpiration rates and a subset of the soil microbial taxa are independently affected by the same abiotic factors or (2) microbes residing in and around the rhizosphere of plants influence plant physiology, or (3) the plant transpiration is influencing the microbial communities in the soil in some way. While we could not distinguish between these hypotheses in the current study, there is accumulating evidence on the associations of microbes with plastic plant phenotypes and physiological processes (Friesen et al., 2011). Some studies find that microbes can influence physiological responses of plants, as microbes can make a unique structural and biochemical pathways available to plants. In a controlled experiment to examine plant soil relations, it is speculated that plants can select for a subset of microbes at different stages of development for different functional purposes (Chaparro et al., 2013). Similarly, studies find that physiological traits of plants can influence the surrounding microbial communities. For example, in a common garden test of spruce seedlings, several groups of fungi and bacteria were significant correlated with a suite of plant physiological traits including transpiration rates (Li et al., 2018), suggesting that these physiological traits can influence associated microbial communities. It is likely that some of the relationships detected were a result of abiotic factors similarly structuring microbial communities and plant transpiration rates and that other relationships were the result of direct interactions between the plants and microbes. Stochastic assembly processes also likely occur, making some groups of microbes weakly related to either plant or abiotic factors. Future experimental manipulations to explore these patterns would be useful in further understanding the functional consequences of plant species selection on water movement in green infrastructure, and how the belowground community matters. Interestingly, the fungi taxa Comamonadaceae and bacteria taxa Chryososporium (Medina et al., 2010) had Pearson correlation values close to 1, across the top three plant water conductors within and across bioswales (Figures 5, 6). Controlled experiments found that species in the Comamondaceae (Li et al., 2015) and Chryososporium host physiological benefits to plants in low quality soils.

Further research is necessary to examine the particular taxa that influence plant transpiration rates in controlled and *in situ* experiments.

Surprisingly, we found low abundance and diversity of AM fungi in soils collected near bioswale plants. This result may be due to the high inputs of nutrient-rich stormwater runoff that is intentionally shunted into bioswale inlets. High nutrient loads are known to decrease AM fungal abundance, as plants allocate less carbon to these belowground symbionts when nutrients are readily available to plant roots (Treseder, 2004). The vast majority of AM fungal OTUs in our dataset were classified as unidentified, highlighting the need for continued research in the taxonomy and identification of AM fungi, even in urban ecosystems. Of those that were identified, the most abundant genera detected in this were *Glomus* and *Rhizophagus*, both of which are cosmopolitan in their distributions and are widely regarded as having generalist life history strategies due to their large genomes and frequent occurrences in disturbed environments (Börstler et al., 2008; Tisserant et al., 2013). Specifically, *Glomus* species are considered ruderal taxa due to their dominance in agricultural and other frequently disturbed soils, fast growth rate and rhizophilic nature (Chagnon et al., 2013; Weber et al., 2019). In past studies, *Glomus* innoculations significantly improved plant transpiration abilities and increased resistance to drought in controlled experiments (Augé, 2004; Siddiqui and Singh, 2004). The high relative abundances of these taxa are consistent with previous findings of AM fungal communities in New York City green roof soils, where *Glomus* and *Rhizophagus* were the most abundant AM fungal taxa detected.

However, the green roof soils harbored significantly higher relative abundances of AM fungi than the present study, which is likely due to the relatively nutrient-limited and stressful environment of green roof communities (McGuire et al., 2013). Of the five plant species we focused on in this study, we detected the highest abundance of AM fungal OTUs associating with *Panicum* species. *Panicum* is a large genus of perennial and annual grasses that are highly dependent on their symbioses with AM fungi for nutrient acquisition, pathogen suppression and tolerance to environmental stressors (Clark et al., 1999). We hypothesized that variation in plant physiology would result in unique assemblages of microbial communities; shifts in the microclimate of individual plants’ rhizospheres due to physical and chemical differences in root structure likely influence assembly of different AM fungi, and plants vary in their dependency on mycorrhizal fungi. The highest abundance of AM fungi detected in the *Panicum* rhizosphere soils may be due to grass species’ tendency to harbor higher loads of AM fungal symbionts; however, we only sampled the soil nearby plants and not the roots themselves and therefore likely only detected AM extra-radical mycelia and spores. Further research investigating the AM fungi colonizing the different plant species roots in addition to the rhizosphere would help unpack the factors contributing to the shifts in AM fungal abundance observed in this study.

## Data Availability Statement

The metagenomic data has been uploaded to the Sequence Read Archive (SRA). Our reference number is PRJNA549763.

## Author Contributions

OB, KM, and MP conceived the ideas and designed the methodology. OB, JH, and SB collected the data. OB, DD, AG, KS, KM, SB, and MP interpreted the results. OB, KM, DD, and KS wrote the manuscript. All authors contributed to the drafts and gave final approval for publication.

## Conflict of Interest

The authors declare that the research was conducted in the absence of any commercial or financial relationships that could be construed as a potential conflict of interest.
